# Calcified multilocular thymic cyst associated with thymoma: a case report

**DOI:** 10.1186/1752-1947-5-225

**Published:** 2011-06-21

**Authors:** Hafsa Elouazzani, Fouad Zouaidia, Ahmed Jahid, Laila Laraqui, Zakiya Bernoussi, Najat Mahassini

**Affiliations:** 1 Department of Pathology, Ibn Sina Hospital, Rabat, Morocco

## Abstract

**Introduction:**

There are few case reports of thymoma with a thymic cyst. Such an association renders it difficult for any pathologist to differentiate from other neoplasms, such as a cystic thymoma.

**Case presentation:**

A 50-year-old Berber woman from Morocco was admitted with a chronic cough of more than 10 years duration. Her medical history and physical examination were normal. Anterior chest radiography demonstrated a calcified opacity in her right anterior mediastinum. A chest-computed tomogram revealed a round cystic tumor, with significant calcification in her right anterior mediastinum. A surgical exploration was performed. The tumor seemed to be a well-encapsulated and totally calcified lesion, arising from the right lobe of her thymus. It was removed by partial resection of her thymus. Through histology, the calcified tumor exhibited some areas of multilocular fibrous-wall cysts. These cysts were partially lined by small cuboidal cells with severe chronic inflammation and an AB thymoma that arose from the wall of the cyst.

**Conclusion:**

Greater attention should be given to multilocular thymic cysts, to exclude the possibility of neoplasm, especially when the cyst wall is thickened.

## Introduction

Multilocular thymic cysts are uncommon lesions of the anterior mediastinum. On a regular histopathology examination, they show significant inflammation and fibrosis that can be associated with thymic neoplasm such as thymoma or thymic carcinoma. It is worth noting that an association between thymoma and multilocular thymic cysts has hardly ever been observed. However, the possibility of other cystic thymic lesions, essentially cystic degeneration of thymoma, must always be considered [1].

We report an unusual AB thymoma case of a 50-year-old woman in the wall of a calcified multilocular thymic cyst. Our study suggests that difficulties related to the diagnosis have been noted, and we advise that special attention be given to every anterior mediastinal cystic lesion.

## Case presentation

**Figure 1:**
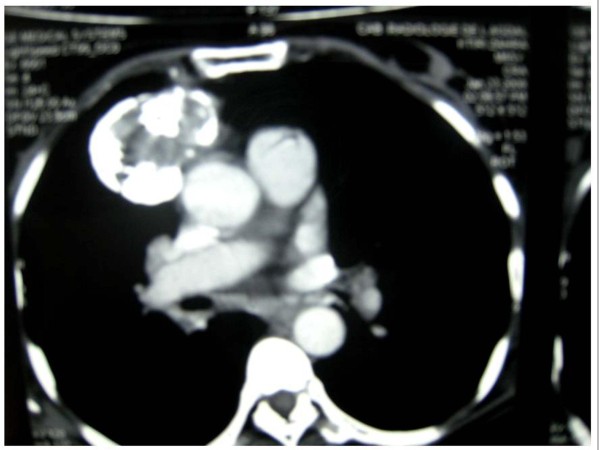
**CT of thorax**. It shows a round cystic tumor, with significant calcification in the right anterior mediastinum.

A 50-year-old Moroccan Berber woman was admitted with a chronic cough that has been going on for more than 10 years, without history of smoking or neoplasm. On admission, her physical examination and routine biochemical tests were within normal limits. Chest radiography showed a round calcified opacity on her right anterior mediastinum. Computed tomography (CT) of her thorax revealed a round cystic tumor, with significant calcification, in her right anterior mediastinum (Figure 1). A surgical exploration revealed a well-encapsulated and totally calcified lesion, arising from the right lobe of her thymus; it was totally removed by partial resection of the thymus.

**Figure 2:**
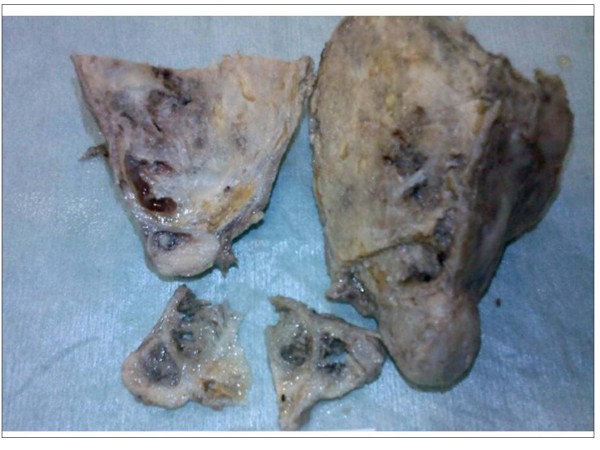
**Surgical specimen**. The calcified tumor was well-encapsulated and showed focally a multilocular thick-wall cysts.

**Figure 3:**
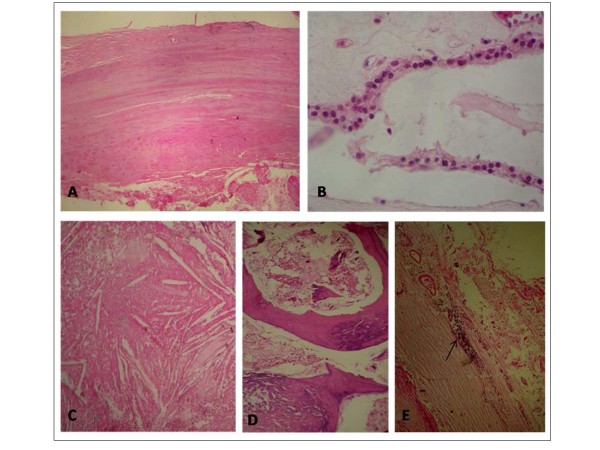
**Photomicrograph**. The fibrous-wall cysts (A), lined partially by small cuboidal cells (B), containing large areas of cholesterol (C), calcification (D) and small foci of lymphoid tissue (E) (hematoxylin-eosin stain ×10).

**Figure 4:**
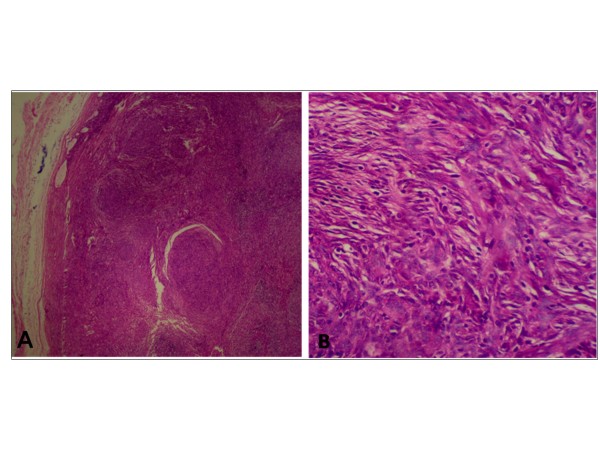
**Photomicrograph**. An AB thymoma arising in the wall of the cyst (A) (hematoxylin-eosin stain ×10), composed of spindle to oval cells with lymphocyte rich areas (B) (hematoxylin-eosin stain ×40).

In a pathological examination of the surgical specimen, the calcified tumor (15 × 8 × 6 cm) was well-encapsulated and focally showed multilocular thick-wall cysts (Figure 2). Histological examination revealed that the fibrous-wall cysts were partially lined by small cuboidal cells, containing small foci of lymphoid tissue with large areas of cholesterol and calcification (Figure 3). In the wall of the cyst we found neoplastic thymic elements composed of spindled to oval cells with lymphocyte rich areas (Figure 4). The pathological diagnosis was an AB thymoma in the pre-existing thymic cyst.

## Discussion

Benign thymic cysts are uncommon lesions that account for approximately 3% of all anterior mediastinal masses. Frequently, they are asymptomatic and the actual identification of the tumor is usually made after surgery and histological examination [1].

Such cysts can either be congenital or acquired in origin. Congenital cysts are typically unilocular. They contain clear fluid and have walls that are thin to the point of translucency; they show no evidence of inflammation on careful histopathological examination [2]. By contrast, acquired thymic cysts result from an inflammatory process. They are usually multilocular, hence the commonly used term "multilocular thymic cyst". The cysts, which contain turbid fluid or gelatinous material, have thick and fibrous walls. Typically, they show evidence of significant inflammation and fibrosis on histopathology examination [3,4].

Multilocular thymic cysts may be associated with thymic neoplasm such as thymoma or thymic carcinoma. They may adhere to adjacent structures and simulate an invasive neoplasm at thoracotomy [5,6].

Multilocular thymic cyst associated with thymoma or a malignant tumor is rare [6,7]. In such cases, the cystic degeneration of a thymoma can be retained. It can result in a gross pathologic appearance that simulates multilocular thymic cyst [8] but histopathological examination reveals a complete absence of epithelial lining within the cystic wall; this is an important feature of cystic thymoma that differentiates it from thymic cyst with thymoma. Indeed, in thymoma, the perivascular spaces are the source of the cystic spaces [8,9].

In the case of thymic cyst with thymoma, the thymoma arises from the wall of a thymic cyst. This consists of a large round mass and small flattened spindle-shaped nodules [8,9]. Because flat and spindle-shaped mural nodules are difficult to observe when using CT and magnetic resonance imaging, it is of vital importance that the histopathologic specimen be carefully inspected to exclude co-existing neoplasia [5,6].

In our case, the calcified pattern hinders the possibility to distinguish a multilocular cyst from cystic thymoma. However, good sampling of the specimen allowed us to see that the wall was lined with epithelial cells, and to therefore and make a diagnosis of multilocular thymic cyst associated with thymoma.

For these lesions, total excision should be performed. This remains the curative treatment of choice, and histological examination is the only definitive means of diagnosis [5,6]. Although thymoma with metastasis or recurrence have rarely been described, in our case the partial resection of the thymus was performed for diagnosis and curative treatment. The post-operative course was uneventful and our patient has had no complications in their two year follow up.

## Conclusion

In suspicious cases of multilocular thymic cysts, thorough sampling of every "thymic cyst" must be carried out. This is not only to establish an accurate diagnosis, but also to exclude the possibility of neoplasm, especially when the cyst wall is thickened.

## Consent

Written informed consent was obtained from the patient for publication of this case report and any accompanying images. A copy of the written consent is available for review by the Editor-in-Chief of this journal.

## Competing interests

The authors declare that they have no competing interests.

## Authors' contributions

HE drafted the manuscript. FZ performed the case management, and drafted the manuscript. AJ participated in confirming the diagnosis. LL participated in the patient's management. ZB and NB corrected the manuscript. All authors read and approved the final manuscript.
